# Enhancing the e-learning system based on a novel tasks’ classification load-balancing algorithm

**DOI:** 10.7717/peerj-cs.669

**Published:** 2021-09-09

**Authors:** Ayman E. Khedr, Amira M. Idrees, Rashed Salem

**Affiliations:** 1 Information Systems Department, Faculty of Computers and Information Technology, Future University in Egypt, Cairo, Egypt; 2 Information Systems Department, Faculty of Computers and Information, Menoufia University, Cairo, Egypt

**Keywords:** Cloud computing, Load balancing, Classification data mining, Students’ satisfaction, E-learning

## Abstract

In the educational field, the system performance, as well as the stakeholders’ satisfaction, are considered a bottleneck in the e-learning system due to the high number of users who are represented in the educational system’s stakeholders including instructors and students. On the other hand, successful resource utilization in cloud systems is one of the key factors for increasing system performance which is strongly related to the ability for the optimal load distribution. In this study, a novel load-balancing algorithm is proposed. The proposed algorithm aims to optimize the educational system’s performance and, consequently, the users’ satisfaction in the educational field represented by the students. The proposed enhancement in the e-learning system has been evaluated by two methods, first, a simulation experiment for confirming the applicability of the proposed algorithm. Then a real-case experiment has been applied to the e-learning system at Helwan University. The results revealed the advantages of the proposed algorithm over other well-known load balancing algorithms. A questionnaire was also developed to measure the users’ satisfaction with the system’s performance. A total of 3,670 thousand out of 5,000 students have responded, and the results have revealed a satisfaction percentage of 95.4% in the e-learning field represented by the students.

## Introduction

E-learning is a term that refers to adapting the information technology tools as well as communication methods in the education sector. E-learning has a positive impact on the education sector for both local and institutional environments. At the institutional level, e-learning provides different tools for students’ managerial aspects as well as educational aspects. The managerial aspects such as students’ enrollment, while the educational aspects such as providing course material and online exams. The local level provides the learning activities for a single course or group of courses. Investing in both levels is essential for ensuring the continuous development in the educational sector which consequently provides continuous development in the whole economy as discussed in Van Hilten (2015) that higher education will continuously have its impact on economic development.

Different success factors are introduced for e-learning including the management process, continuous development, the level of quality of the system infrastructure, and the system’s continuous availability and reliability (McGill, Klobas & Renzi, 2014). As discussed in Sultan et al. (2017) and Al Mazroi, Khedr & Idrees (2021), the system’s success is measured by the user satisfaction level through the high quality of the system and the availability of the system’s usage. As discussed in Van Hilten (2015), one of the main challenges of e-learning success is overcoming the infrastructure issues which is a key factor for the e-learning environment. Infrastructure is a basic requirement for the successful access of e-learning tools. Fernández et al. (2012) presented a situation in a “machine learning course at Stanford that accepted over 160,000 students as applicants. The high number of students emphasized the strong requirement of a reliable infrastructure that clearly exceeded the capability of a conventional server. This demand for a highly reliable infrastructure resulted in the failure of the required activities and requests, especially in peak time. This situation highlighted the impact of cloud computing on e-learning systems.

Through many researches, cloud computing has been introduced to be a solution for higher resources’ utilization than conventional servers. With its elasticity, fast, scalability, and flexibility features, cloud computing has been introduced as an innovative direction of empowering IT solutions. Different benefits can be obtained when applying the e-learning system on the cloud platform, the following can summarize some of these benefits: One of the main benefits is ensuring the enhancement of the e-learning system’s performance as the system’s services will be deployed on the cloud platform. Deploying the system’s services on the cloud computing platform also leads to the reduction of the system’s cost such as maintenance and administration cost. Another benefit is the continuous availability of the system’s services for the system’s stakeholders. The students and teachers can use the services from different places using different devices.

One of the main features of cloud computing is flexibility. Therefore, e-learning systems can be mounted as required to ensure maintaining the required level of investment (Mostafa et al., 2020). The continuous availability of up-to-date software is also one of the vital benefits that have a positive impact on e-learning systems. Although cloud computing has its high positive impact on e-learning systems, however, cloud computing may face different challenges such as maintaining data, system security, ensuring load balancing among the system’s working nodes, providing the availability for data backup and portability, support the multiple platforms, and ensure the system’s reliability (Kalapatapu & Sarkar, 2012).

Load balancing is defined as the process of improving the resources’ utilization targeting to effectively enhance the tasks’ response time (Ramana, Subramanyam & Ananda Rao, 2011). This improvement is reached through re-distributing the load among the system’s nodes. Load balancing ensures the balance in the load that is provided to the systems’ nodes in order to avoid overloading one node while under-loading another. Different load balancing algorithms are introduced for reaching the required target. These algorithms will be presented in “Related Work”. Load balancing algorithms are evaluated in cloud computing through the measurement of different criteria. These criteria are throughput, overhead, fault tolerance, response time, complexity, performance, scalability, resource utilization, speed, overhead, power consumption and waiting time. These criteria are interrelated, which leads to the effect of one criterion on the other. For example, response time, speed, and waiting time have a direct impact on performance.

The remaining of the paper includes a brief background in “Background” and the related work is discussed in “Related Work”. The proposed approach is discussed in detail in “E-Learning Based on Task Classification Load Balancing Algorithm” while the evaluation measures are discussed in “Evaluation Measures Applied in the Case Studies”. The experiments are then illustrated in “Simulation Case Study” and “Real Case Study Applied in E-Learning System and Experimental Results” with discussing these results in “Conclusion”. Finally, the conclusion is discussed in “Conclusion”.

## Background

Load balancing algorithms follow one of the two approaches, static and dynamic (Elmasry, Khedr & Nasr, 2019). Algorithms that follow the static approach need to determine the system’s capabilities such as the system’s resources and the required communication time (Khedr, Idrees & Alsheref, 2019). The algorithms that follow the static approach apply the round-robin algorithm, which is a simple technique with low resource utilization. However, this algorithm does not guarantee equity in the load distribution as the current status of the servers is not considered (Chen, Chen & Kuo, 2016). This situation highlighted that static algorithms are not suitable for cloud systems.

On the other hand, the main concept of the dynamic approach is the continuous monitoring of the current system’s status. This leads to the possibility of the tasks’ migration from one node to another based on the current status of these nodes. One of the considerations of dynamic algorithms is complexity. However, this was not highlighted by many researchers due to other benefits such as the high performance and accuracy in the load distribution for the system’s nodes (Kanakala, Reddy & Karthik, 2015). Different researches followed the dynamic approach such as (De Falco et al., 2015). The proposed approaches considered scheduling the tasks based on the system’s status. However, the high cost and eliminated considerations of different vital factors were noticed.

Different load balancing algorithms have been proposed such as in Afzal & Abdur Rahman (2020) which proposed a load balancing approach that aimed at minimizing the whole task’s executing time and reached a satisfactory result. However, it did not consider the migration time of the task through the network. Earlier, another research in Afzal & Kavitha (2018b) considered the migration process by proposing a load balance algorithm based on migrating tasks to the less loaded machines with minimum migration cost. The proposed algorithm succeeded in its objective. However, the current research has an advancement for direct allocation to the required machines with no need for migrating from machines which ensures less total execution time. Finally, the research in Afzal, Kavitha & Gull (2020) discussed the relation between different factors including the number of servers, number of tasks, and different time parameters such as waiting time and response time. The research concluded that following the parallel approach is satisfying when the allocation rate is higher than the tasks’ arrival rate. The research also concluded that the relationship between the waiting time and the required tasks is a linear relation. In the current research, the parallel approach is followed with overcoming the negative aspects in the concluded relationships.

On the other hand, different evaluation metrics for load balance algorithms have been introduced. The research in Afzal & Kavitha (2018a) presented the evaluation metrics in a hierarchical representation. The metrics were divided into performance and economic metrics while the second level for both branches was qualitative and quantitative following by dependent and independent metrics’ classification. In the current research and according to the classification presented in Afzal & Kavitha (2018a), evaluating the proposed algorithm followed the performance plan and covered both the dependent and independent quantitative directions. The research in Afzal & Kavitha (2018a) was followed by Afzal & Kavitha (2019) which presented load balance algorithms classification hierarchy. The classification included two main branches, they are scheduling and load allocation. According to the presented hierarchy, the proposed algorithm in the current study follows the scheduling branch. It also has an advance over the proposed classification as it considers memory, CPU, server load and task characteristics.

## Related work

The high impact on the e-learning systems has attracted many researchers to introduce different research in this field (Hassouna et al., 2020). Different educational organizations targeted to deploy their e-learning systems over cloud computing platforms such as in Angelova, Kiryakova & Yordanova (2015). Focusing on load balancing algorithms, this section presents different research that has been introduced in applying load balancing algorithms over the e-learning systems that are deployed on the cloud computing platform.

As argued by Mihaescu et al. (2011), most of the current e-learning systems are deployed on a determined machine with no ability for distributing its infrastructure’s components. The research in Mihaescu et al. (2011) highlighted that this situation raises a serious issue of the e-learning systems’ scalability which directly affects its performance. Therefore, the load balancing benefits allowed the researchers to be highly motivated for introducing load balancing algorithms as a solution. Mihaescu et al. (2011) presented an infrastructure for an e-learning system that was based on applying the load balancing paradigm based on weighting the required service. The system has been applied to a simulation tool. The introduced system included a fixed set of servers with no ability for extending this set, this issue was one of the authors’ future works. The research also did not provide a clear description of the load balancing methodology as well as the tasks’ weighting approach.

Another research (Rajarajeswari, 2013) introduced an approach for load balancing in e-learning systems. The proposed approach depended on applying a clustering technique to cluster the working nodes, then estimating their workload is performed. Based on the determined workload for each node, the required tasks are then distributed to the less-loaded nodes. The proposed approach applies the k-means algorithm for clustering nodes without introducing any proof of the suitability of this algorithm for the applied characteristics. The research also did not consider the variations of the required tasks in the distribution process. Finally, the proposed load balancing approach in Rajarajeswari (2013) has not been compared with other well-known algorithms.

Recently, Dominic & Francis (2014) proposed a load-balancing approach in e-learning. The proposed approach adopted the concept of developing a hashing table with the nodes which store the task data. The data stored in the hashing table is then used in building a binary tree for connecting all working tasks. This binary tree is the main source for identifying the available servers. Based on the discussion of the research, the binary tree is the main step for minimizing the search time to respond to the required tasks. The implementation and evaluation of the proposed approach have not been introduced with highlighting that it was one of the future work for the authors in addition to the required explanation of the binary tree contribution. More recently, Khedr & Idrees (2017a) proposed a comparison of two load balancing algorithms targeting to propose the suitable model to enhance the educational process. The research introduced an experimental result that revealed the advancement of the load balancing algorithm “task scheduling algorithms” over the “Random Allocation Load balancing” in the educational field. The authors highlighted that more research is required to include different parameters such as power consumption and different network load situations.

Finally, a study by the same authors (Khedr & Idrees, 2017b) presented the impact of applying the e-learning system on the cloud environment. The research focused on the students’ satisfaction level in one of the Egyptian universities through enhancing the system’s performance. Applying the enhanced system has revealed students’ satisfaction level equal to 89.7%. As the previously discussed research has revealed different limitations, this study aimed to prove the applicability of the proposed algorithm by comparing the algorithm with some of the previously proposed algorithms. The evaluation included a set of standard metrics.

## E-learning based on task classification load balancing algorithm

In this section, a task classification load balancing algorithm (TCLB) is proposed. The main target for the proposed task is to efficiently allocate the users’ requests on the cloud server nodes. The efficient allocation ensures the highest utilization of the cloud nodes and the highest throughput with the lowest response time. Efficient distribution of the workload is achieved through applying a mining technique as a preparatory phase in order to classify the users’ requests and estimate the resources’ consumption by the network tasks. The classification phase is based on different criteria such as tasks’ memory usage and CPU utilization. Classifying the workload is a key phase to successfully determine the appropriate cloud node for execution. The following sub-sections describe the algorithm architecture, the main phases, and the pseudo-code of the proposed algorithm.

The main contribution of the proposed algorithm can be summarized as follows:

1. Optimizing the cloud node selection for executing a determined user’s request based on applying mining techniques. A classification algorithm is applied to select the best cloud node for executing the determined task. Successful selection of the cloud node ensures the optimization for the whole execution process of the system.
2. Maintaining the pre-executed tasks repository is performed. This repository is considered a historical resources repository. It is used to estimate the required resources for the new users’ tasks by searching for similar tasks in this repository. In case that there were no similar tasks in the repository, then applying the classification step is performed. This procedure minimizes the required estimation time for the tasks as it ensures that the classification phase is only fired in case that the required task is not previously performed by other users.
3. Continuous enriching of the pre-executed tasks repository ensures a continuous enhancement in the classification process, which is one of the main steps towards optimization.

### The proposed e-learning system architecture

**Figure 1:**
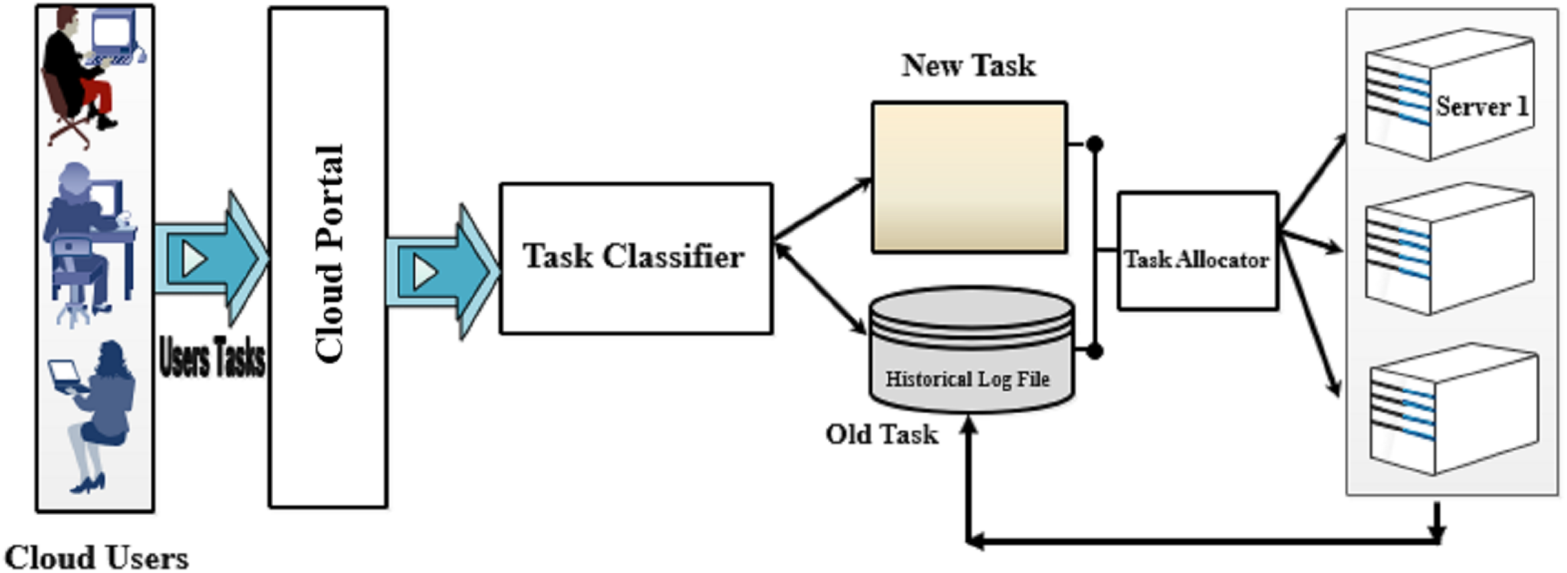
The proposed e-learning system architecture.

The task classification load balancing algorithm includes two main phases namely “Task Classifier”, and “Task Allocator” which are illustrated in Fig. 1. The task classifier phase focuses on the determination of the task requirements by applying a classification algorithm to discover the group that the task belongs to. The task classifier phase receives its input from the cloud portal which gathers all the users’ requests and classifies them. The output of the task classifier is then inserted in the tasks’ pool with the required information. Then the task allocator phase is responsible for allocating the tasks to the suitable server based on the proposed technique which will be discussed in detail.

### Description of the proposed e-learning system’s main phases

The detailed steps of each phase are described in this section in two perspectives. First, the basic functions’ steps are demonstrated to highlight the general process of each phase as well as the formal description of the main algorithm’s components. Then, the pseudo-code is illustrated to present the algorithm’s detailed steps.

#### Phase 1: task classifier phase

The main outcome of the “Task Classifier Phase” is the successful estimation of the resources’ requirements for the users’ requests. This outcome is reached by classifying the users’ requests to the most representative classes. Each class is represented by a set of features that map to the task’s description as well as the system’s description. The input of “Task Classifier Phase” is the set of the users’ requests that reside in the cloud portal pool. The cloud portal pool (CP) that includes the users’ requests is formally represented as a set of tasks from T_1_ to T_n_ by formula 1.

(1) $${\rm{CP}} = \{ {{\rm{T}}_1},{{\rm{T}}_2}, \ldots ,{{\rm{T}}_{\rm{n}}}\} \>|\>{\rm{n}}\> \in \>{\rm{N}},\>{\rm{n}}\>{\rm{is}}\>{\rm{the}}\>{\rm{number}}\>{\rm{of}}\>{\rm{tasks}}$$

The main outcome of the “*Task Classifier Phase*” is reached by applying three main steps, they are the *Primary data preparation* step, the *task clustering step*, and the *task classification step*. The following subsections provide a detailed description of each step.

— Primary Data Preparation Step

The first step is to prepare the primary data which is essential for initializing the algorithm. This primary data is represented in a set of tasks that are described by a set of parameters. The parameters’ set includes two categories of elements. The first category represents the task such as the required CPU utilization and the required memory utilization. While the second category represents the server such as the server’s response time and latency. On the other hand, the primary data can be obtained by two methods. One of these methods is simulation-based in which random requests are generated, then these requests are performed on the system’s server and the required parameters are measured. The second method is using the pre-performed tasks in the network as the description of these tasks resides in the tasks’ log pool.

The outcome of this step is presented as a set named (LogReq) that includes a group of vectors. Each vector represents a task associated with its parameters’ values. For more clarification, the log file set (LogReq) is the set of all tasks that are previously served in the network with their associated parameters’ values. The log file set members are represented as vectors. This vector representation has a total of eight elements. The first element is the task identifier T_*i*_ and the remaining seven elements describe the performance measurements of the server as well as the task on focus. These eight measurements are the throughput (TTH), response time (TRT), processor utilization (TPU), memory usage (TMU), bandwidth utilization (TBU), latency (TL), error rate (TER), reliability (TREL). The previous description of the log file set (LogReq) is formally represented by formula 2 and is considered the main input of the next step “*clustering step*”.

(2) $${\rm{LogReq}} = \{ \left\langle {{{\rm{T}}_{\rm{i}}},{\rm{TT}}{{\rm{H}}_{\rm{i}}},{\rm{TR}}{{\rm{T}}_{\rm{i}}},{\rm{TP}}{{\rm{U}}_{\rm{i}}},{\rm{TM}}{{\rm{U}}_{\rm{i}}},{\rm{TB}}{{\rm{U}}_{\rm{i}}},{\rm{T}}{{\rm{L}}_{\rm{i}}},{\rm{TE}}{{\rm{R}}_{\rm{i}}},{\rm{TRE}}{{\rm{L}}_{\rm{i}}}} \right\rangle |\>{\rm{i}}\> \in \>{\rm{N}}\} $$

— Tasks’ Clustering Step

In this step, a clustering algorithm is applied for grouping the generated tasks. An extensive review is performed in order to determine the suitable algorithm to be applied. Xu & Wunsch (2005) have performed a survey on clustering algorithms. The survey discussed that the clustering algorithms are following two approaches; they are partitioning and hierarchical. As the current data is not hierarchical in nature, therefore, the study should follow the partitioning approach. Focusing on the partitioning approach, Xu & Wunsch (2005) presented the k-means algorithm and its enhancements. The study revealed that k-means had the advantage of its computational simplicity. This simplicity is reflected in the low time complexity which is equal to O(nct) where n is the number of iterations, *c* is the number of classes, and t is the number of tasks. Another research that was presented by Xu & Tian (2015) confirmed the advantages of using the k-means algorithm due to its suitability for large datasets in addition to its simplicity in implementation.

One of the main drawbacks of k-means was the random determination of the initial point due to the optimization of the non-convex data which was also highlighted in Lindsten, Ohlsson & Ljung (2011). This drawback was further considered in a research conducted by Khedr, El Seddawy & Idrees (2014). The research proposed that determining the centroid initial point instead of the random initial cluster point is more accurate. Another consideration is the requirement of pre-determining the suitable number of clusters as this affects the data distribution to the clusters. However, this was not a bottleneck in this research, as it is determined that the number of clusters will be equal to the number of servers of the network.

For more clarification, the main objective of the research is the uniform distribution of the required tasks to ensure equal load for all participating servers in the available operating time. The research follows the partitioning approach, not the hierarchical approach, which also led to the ability for a one-level partitioning. Therefore, the number of clusters was set to be equal to the number of available servers. This decision has been reached for ensuring the implementation simplicity without the need to further allocating different clusters’ members to the same server.

The set of classes (C) associated with the parameters describing the class is formally represented by formula 3. Moreover, the set of vectors representing the classes associated with their members; which are the clustered users’ requests (CLustT); is formally represented by formula 4.

The set of tasks’ classes (C) include a set of vectors that equal to the number of classes, the elements of each vector are the class name (Ca) and the set of performance parameters that describe this class. These eight measurements are the throughput (CTH), response time (CRT), processor utilization (CPU), memory usage (CMU), bandwidth utilization (CBU), latency (CL), error rate (CER), reliability (CREL)

(3) $${\rm{C}} = \{ \left\langle {{{\rm{C}}_{\rm{a}}},{\rm{CT}}{{\rm{H}}_{\rm{a}}},{\rm{CR}}{{\rm{T}}_{\rm{a}}},{\rm{CP}}{{\rm{U}}_{\rm{a}}},{\rm{CM}}{{\rm{U}}_{\rm{a}}},{\rm{CB}}{{\rm{U}}_{\rm{a}}},{\rm{C}}{{\rm{L}}_{\rm{a}}},{\rm{CE}}{{\rm{R}}_{\rm{a}}},{\rm{CRE}}{{\rm{L}}_{\rm{a}}}} \right\rangle |\>{\rm{a}}\> \in \>{\rm{m}}\} $$

where: m is the number of classes

The set of clustered tasks (ClustT) is also represented as a set of vectors. The elements of each vector are the class name proceeded by the tasks that are members of this class. The number of elements in each vector depends on the number of tasks that belong to each class.

(4) $${\rm{CLustT = }}\{ \lt {{\rm{C}}_{\rm{b}}},{{\rm{T}}_{\rm{s}}}, \ldots ,{{\rm{T}}_{\rm{v}}} \gt \} \;{\rm{|}}\;{\rm{b}}\; \in \;\{ {\rm{1}},{\rm{2}}, \ldots ,{\rm{f}}\} ,{\rm{s}},{\rm{y}}\; \in \;\{ {\rm{1}},{\rm{2}}, \ldots {\rm{n}}\}$$

where: ∃ C_a_ = C_b_ such that < C_a ,_ CTH_a_, CRT_a_, CPU_a_, CMU_a_, CBU_a_, CL_a_, CER_a_, CREL_a_ **>** ∈ C, f number of classes, and n number of tasks

The outcome of this step is the training data for the next step “*Tasks’ classification step*” which produces the main phase outcome.

— Tasks Classification Step

In this step, classifying the users’ requests that exist in the cloud portal pool is applied. The main aim of this step is to determine an accurate estimation of the attributes which describe the user’s request. The main outcome of this step is to describe each task with the required resources to accomplish this task in addition to the predicted performance of the server that is executing the task. This outcome will further be considered in the *task allocation* phase as a critical input to successfully distribute the tasks in the servers’ pool with ensuring high performance.

A survey was presented in Abd AL-Nabi & Ahmed (2013) which highlighted the advantages of the decision tree algorithms for classification. These advantages were the lower computation time which is O(m· n) compared with other classification techniques’ categories where m is the number of records and n is the number of attributes (Esmeir & Markovitch, 2007). One of the efficient decision tree algorithms is ID3 (Khedr, Idrees & El Seddawy, 2016a), whose advantage is its ability to deal with noise data. However, as illustrated in Abd AL-Nabi & Ahmed (2013) and other researches such as in Pirdavani et al. (2015), the main drawback of the decision tree algorithms is the high cost in building the tree with the positive relationship between the number of clusters and the error rate.

This research applied the enhanced ID3 algorithm which was presented in Khedr, Idrees & El Seddawy (2016a) targeting to hinder the drawback. Khedr, Idrees & El Seddawy (2016a) engaged the concept of data partitioning which consequently revealed the opportunity for parallelism in applying the ID3 algorithm on the data subsets simultaneously. The proposed approach adopted the examination of the data subsets with setting the decision of examining the data element for the classification task within its siblings with no consideration to other data elements. This approach revealed the opportunity for higher performance while maintaining the classification accuracy level. The enhancement in the research of Khedr, Idrees & El Seddawy (2016a) succeeded to overcome the limitations of the decision tree algorithms. Khedr, Idrees & El Seddawy (2016a) proved its applicability in different domains by applying two experiments with different characteristics in two domains. They are banking and radiology data. The classified users’ requests (CLassT) are represented as sets of vectors representing the classes that are associated with their members. The formal representation for CLassT is in formula 5. The elements of each vector include the class identifier as the first member in the vector followed by the tasks that are classified as members of the class. The number of elements in each vector depends on the number of the classified tasks of each class.

(5) $${\rm{CLassT = }}\left\{ {{{ \lt }}{{\rm{C}}_{\rm{b}}}{\rm{, }}{{\rm{T}}_{\rm{v}}}{\rm{, \ldots , }}{{\rm{T}}_{\rm{w}}}{{ \gt }}} \right\}{\rm{ | b }} \in {\rm{ }}\left\{ {{\rm{1,2, \ldots , f}}} \right\}{\rm{, v,w }} \in {\rm{ }}\left\{ {{\rm{1,2, \ldots n}}} \right\}$$

where: ∃ C_a_ = C_b_ such that < C_a ,_ CTH_a_, CRT_a_, CPU_a_, CMU_a_, CBU_a_, CL_a_, CER_a_, CREL_a_ >, f is the number of classes, and n is the number of tasks

These results present the tasks’ membership in a determined class. Based on this classification, the required resources for each task are estimated based on the average requirements for all the members in the same class. This assumption is applied based on the study presented by Dahab et al. (2010) which confirmed a claim targeting the relation between siblings. Dahab et al. (2010) claimed that the siblings normally act in the same manner. This claim was further confirmed by a research presented in Khedr et al. (2017) which is applied in a different field and proved the applicability of the assumption in general. Therefore, this study determines the required resources by applying the same concept in estimating the required resources for a determined task based on its class membership.

#### Phase 2: task allocator phase

The basic function steps of the *task Allocator* phase can be discussed as follows:

First, the classified users’ requests set members “CLassT” are determined, the “CLassT” set is previously described in formula 5. Then, determining the available servers set members “AvailServers” is performed through selecting the servers whose availability status is below the threshold. Finally, allocating the users’ requests to their most suitable servers is applied. The set of servers (ServList) is formally described in formula 6 which represents the servers with the terms (S_1_ to S_t_) where t is the number of servers. Moreover, the set of available servers (AvailServ) is formally described in formula 7 which is a subset of the servers set (ServList) and represents the servers which availability status is below the threshold. This means that the servers are available for more tasks’ allocation.

(6) $${\rm{ServList = }}\left\{ {{{\rm{S}}_{\rm{1}}}{\rm{, \ldots }}{{\rm{S}}_{\rm{t}}}\;{\rm{|\; t }} \in {\rm{ N}}} \right\}$$

where: t is the number of servers in the system

(7) $${\rm{AvailServ = }}\left\{ {{{\rm{S}}_{\rm{h}}}\;{\rm{|\; h }} \in {\rm{ }}\left( {{\rm{1,2,}}..{\rm{ t}}} \right)} \right\}$$

where: h is the number of available servers in the system, AvailServ ⊆ ServList, S_h_ ∈ ServList

The allocation idea is to determine the total availability percentage in the network as well as the total requests’ resources estimation. The allocation is performed in two steps. The first step is to allocate tasks to servers which are above the minimum availability threshold of a server j. Then the second step is to continue allocating the remaining requests to the servers with respect to the minimum availability threshold. The availability threshold is maintained to ensure the high performance of the system. As discussed by Khedr, Kholeif & Hessen (2015), the allocation of all the resources of a server leads to the degradation of the server’s performance. Therefore, maintaining a minimum allocation threshold is one of the key factors of maintaining the system’s performance.

The term (TY) refers to the task class. Each class is characterized by a set of parameters that describe the effect of the task on the server status. These parameters are the server’s throughput (CTH), response time, (CRT), CPU utilization (CPU), memory usage (CMU), bandwidth utilization (CBU), latency (CL), and error rate (CER). These parameters are determined based on the task class. As discussed earlier, each task is assigned to a determined class, therefore, the values of these parameters are the class description that the task belongs to. Determining the task type is a key factor in estimating the parameters which will determine the estimated server’s performance.

The type (TY) of a task j can be described as a vector of seven elements as follows:

(8) $${\rm{T}}{{\rm{Y}}_{\rm{j}}} \lt ={\rm{ CT}}{{\rm{H}}_{\rm{q}}}{\rm{, CR}}{{\rm{T}}_{\rm{q}}}{\rm{, CP}}{{\rm{U}}_{\rm{q}}}{\rm{, CM}}{{\rm{U}}_{\rm{q}}}{\rm{, CB}}{{\rm{U}}_{\rm{q}}}{\rm{, C}}{{\rm{L}}_{\rm{q}}}{\rm{, CE}}{{\rm{R}}_{\rm{q}}} \gt {\rm{ | j }} \in {\rm{ }}\left\{ {{\rm{1, \ldots }}{\rm{.n}}} \right\}{\rm{, q }} \in {\rm{ }}\left\{ {{\rm{1, \ldots }}{\rm{.c}}} \right\}$$

where: ∃a = q s.t **<** C_a ,_ CTH_a_, CRT_a_, CPU_a_, CMU_a_, CBU_a_, CL_a_, CER_a_, CREL_a_ **>** ∈ C

n number of tasks, c number of classes

The requirements (TL) of a task (T) are characterized by three parameters, they are: the required CPU utilization for task T (TPU), the required memory usage for task T (TMU), and the required bandwidth usage for the task T (TBU). These parameters are determined based on the task type (TY).

(9) $${\rm{T}}{{\rm{L}}_{\rm{j}}}{\rm{ = T}}{{\rm{Y}}_{\rm{j}}}$$

— Calculate the Total Current Load of the Working Server

The total current load (TCL) of a determined server at the allocated time (t) is identified by the total consumed percentage of the three parameters CPU utilization, memory usage, and bandwidth.

Therefore, the set of tasks allocated to a server i (TS_i_) can be described as follows:

(10) $${\rm{T}}{{\rm{S}}_{\rm{i}}}{\rm{ = }}\left\{ {{{\rm{T}}_{\rm{f}}}{\rm{, \ldots }}{\rm{.}}{{\rm{T}}_{\rm{e}}}{\rm{| f, e }} \in {\rm{ }}\left( {{\rm{1, \ldots }}{\rm{.n}}} \right)} \right\}$$

where i is the server id, n is the number of tasks in the cloud portal. T_f_, T_e_ ∈ CP

The total current load (TCL) of the server i can be described in formula 11 as a vector representing the server’s current processor utilization, memory usage, and bandwidth usage. This vector can be further explained as each member represents the accumulation of the usage for the server’s included tasks. Formulas 12, 13, and 14 represent the accumulation of processor utilization (PU), memory usage (MU), and bandwidth utilization (BU) respectively.

(11) $${\rm{TC}}{{\rm{L}}_{\rm{i}}} = \lt {\rm{ P}}{{\rm{U}}_{\rm{i}}}{\rm{, M}}{{\rm{U}}_{\rm{i}}}{\rm{, B}}{{\rm{U}}_{\rm{i}}} \gt {{ }}$$

(12) $$\rm P{U_i} = {\rm{ }}P{U_i} + {\scriptstyle{\sum}} \;{\rm{ }}TP{U_j}|{\rm{ }}j{\rm{ }} \in {\rm{ }}\left\{ {1, \ldots .n} \right\},{\rm{ }}i{\rm{ }} \in {\rm{ }}\left\{ {1, \ldots .m} \right\},{\rm{ }}{T_j} \in {\rm{ }}T{S_i},$$

(13) $$\rm M{U_i} = {\rm{ }}M{U_i} + {\scriptstyle{\sum}} \;{\rm{ }}TM{U_j}|{\rm{ }}j{\rm{ }} \in {\rm{ }}\left\{ {1, \ldots .n} \right\},{\rm{ }}i{\rm{ }} \in {\rm{ }}\left\{ {1, \ldots .m} \right\},{\rm{ }}{T_j} \in {\rm{ }}T{S_i},$$

(14) $$\rm B{U_i} = {\rm{ }}B{U_i} + {\scriptstyle{\sum}} \;{\rm{ }}TB{U_j}|{\rm{ }}j{\rm{ }} \in {\rm{ }}\left\{ {1, \ldots .n} \right\},{\rm{ }}i{\rm{ }} \in {\rm{ }}\left\{ {1, \ldots .m} \right\},{\rm{ }}{T_j} \in {\rm{ }}T{S_i},$$

where: n number of tasks, m number of servers, TS_i_ is the set of tasks working for the server i

— Calculate the Available Resources of the Working Server

The available resources (ARes) of a determined server j at the allocated time are calculated by determining the remaining percentage of the three parameters: CPU utilization, memory usage, and bandwidth utilization. Therefore, the available resources (ARes) of server j are described as a vector of three elements as in formula 15. The three vector elements are further explained as each member represents the accumulation of the server availability. Formulas 16, 17, and 18 represents the accumulation of the processor utilization (APU), accumulation of memory usage (AMU), and accumulation of bandwidth utilization (ABU) respectively.

(15) $$\rm ARe{s_j} = {\rm{ }} \lt AP{U_j},{\rm{ }}AM{U_j},{\rm{ }}AB{U_j} \gt$$

(16) $$\rm APUj{\rm{ }} = {\rm{ }}SPUj{\rm{ }} - {\rm{ }}PUj{\rm{ }}|{\rm{ }}j{\rm{ }} \in {\rm{ }}\left\{ {1, \ldots .n} \right\},{\rm{ }}i{\rm{ }} \in {\rm{ }}\left\{ {1, \ldots .m} \right\}$$

(17) $$\rm AM{U_j} = {\rm{ }}SM{U_j} - {\rm{ }}M{U_j}|{\rm{ }}j{\rm{ }} \in {\rm{ }}\left\{ {1, \ldots .n} \right\},{\rm{ }}i{\rm{ }} \in {\rm{ }}\left\{ {1, \ldots .m} \right\}$$

(18) $$\rm AB{U_j} = {\rm{ }}SB{U_j} - {\rm{ }}B{U_j}|{\rm{ }}j{\rm{ }} \in {\rm{ }}\left\{ {1, \ldots .n} \right\},{\rm{ }}i{\rm{ }} \in {\rm{ }}\left\{ {1, \ldots .m} \right\}$$

— Calculate the Server’s Working Performance

The working performance (WP) is calculated by three parameters, they are CPU utilization (PU), memory usage (MU), and server bandwidth (BU). As discussed earlier in this section, each parameter has its weight (WPU, WMU, WBU) for the same parameters respectively, which measure the importance of the parameter in estimating the server’s performance.

The working performance (WP) of a server i is calculated as presented in formula 19:

(19) $$\rm WPi{\rm{ }} = {\rm{ }}\left( {PUi{\rm{ }} \times {\rm{ }}WPU{\rm{ }} + {\rm{ }}MUi{\rm{ }} \times {\rm{ }}WMU{\rm{ }} + {\rm{ }}\left( {BUi{\rm{ }}/{\rm{ }}TBU{\rm{ }} \times {\rm{ }}100} \right){\rm{ }} \times {\rm{ }}WBU} \right){\rm{ }}/{\rm{ }}3$$

where TBU is the total network bandwidth.

Then, calculating the network total performance (TP) is formally illustrated in formula 20.

(20) $${\rm TP} = \sum\nolimits_{(i = 1)}^m {{P_i}/m} $$

where p represents the performance and m is the number of servers

— Calculating the Weight of the Server

The weight of a server (W) is calculated by determining the percentage of the server’s performance to the total servers’ performance in the network.

The weight (W) of a server i is calculated as in formula 21:

(21) $$\rm {W_i} = {\rm{ }}{P_i}/{\rm{ }}TP{\rm{ }} \times {\rm{ }}100 \;{{ Where}}\,{\rm{TP}}\,{\rm{is}}\,{\rm{the}}\,{\rm{total}}\,{\rm{network}}\,{\rm{performance}}$$

— Calculate the Estimated Performance of the Server

The estimated performance (EP) of the server is related to the total estimated utilization (TEP) of all tasks that are planned to be allocated on the server and the working performance of the server (WP). The parameters describing the tasks’ performance are the server’s throughput (CTH), the response time (CRT), CPU utilization (CPU), memory usage (CMU), bandwidth utilization (CBU), latency (CL), and error rate (CER). As discussed earlier in this section, each parameter has its associated weights (WCTH, WCRT, WCPU, WCMU, WCBU, WCL, and WCER) which contribute to estimating the server’s performance.

The estimated utilization (TEP) of a task i can be described as in formula 22:

(22) $$\eqalign{\rm TEPi\, = \,\left( {CT{H_i}/\,TTH\, \times \,100} \right)\, \times \,WCTH\, + \,\left( {CR{T_i}/TRT\, \times \,100} \right)\, \times \,WCRT\, \cr\quad + \,CP{U_i}\, \times \,WCPU\, + \,CM{U_i}\, \times \,WCMU\, + \,\left( {CB{U_i}/TBU\, \times \,100} \right)\, \times \,WCBU\, \cr\quad + \,\left( {C{L_i}/TL\, \times \,100} \right)\, \times \,WCL\, + \,CE{R_i}\, \times \,WCER)\,/\,7}$$

where TTH is the total throughput of the network, TRT is the total response time of the network, TL is the total latency of the network.

The estimated allocation performance (EAP) of the server j is related to the estimated utilization (TEP) of all tasks that are planned to be allocated on the server j

— The set of allocated tasks to a server can be formally described as in formula 23:

(23) $$\rm AllocTj{\rm{ }} = {\rm{ }}\left\{ {Sr, \ldots sd{\rm{ }}\left| {r,d{\rm{ }}\left( {1,2,..,{\rm{ }}n} \right),{\rm{ }}n{\rm{ }} = {\rm{ }}} \right|CP|} \right\}$$

— The estimated allocation performance (EAP) of the server i can be described as in formula 24:

(24) $$\rm EAPj{\rm{ }} = {\scriptstyle{\sum}} \;{\rm{ }}TEPi{\rm{ }}/{\rm{ }}\left| {STj} \right|$$

where:|STj| = n (the number of elements in the set of allocated tasks for server j), i ∈ {1,….n}

— Finally, the estimated performance (EP) of the server i can be described as in formula 25:

(25) $$\rm EPj{\rm{ }} = {\rm{ }}\left( {WPj{\rm{ }} + {\rm{ }}EAPj} \right){\rm{ }}/{\rm{ }}2$$

### The pseudo-code of task classification load balancing algorithm

**Figure 2:**
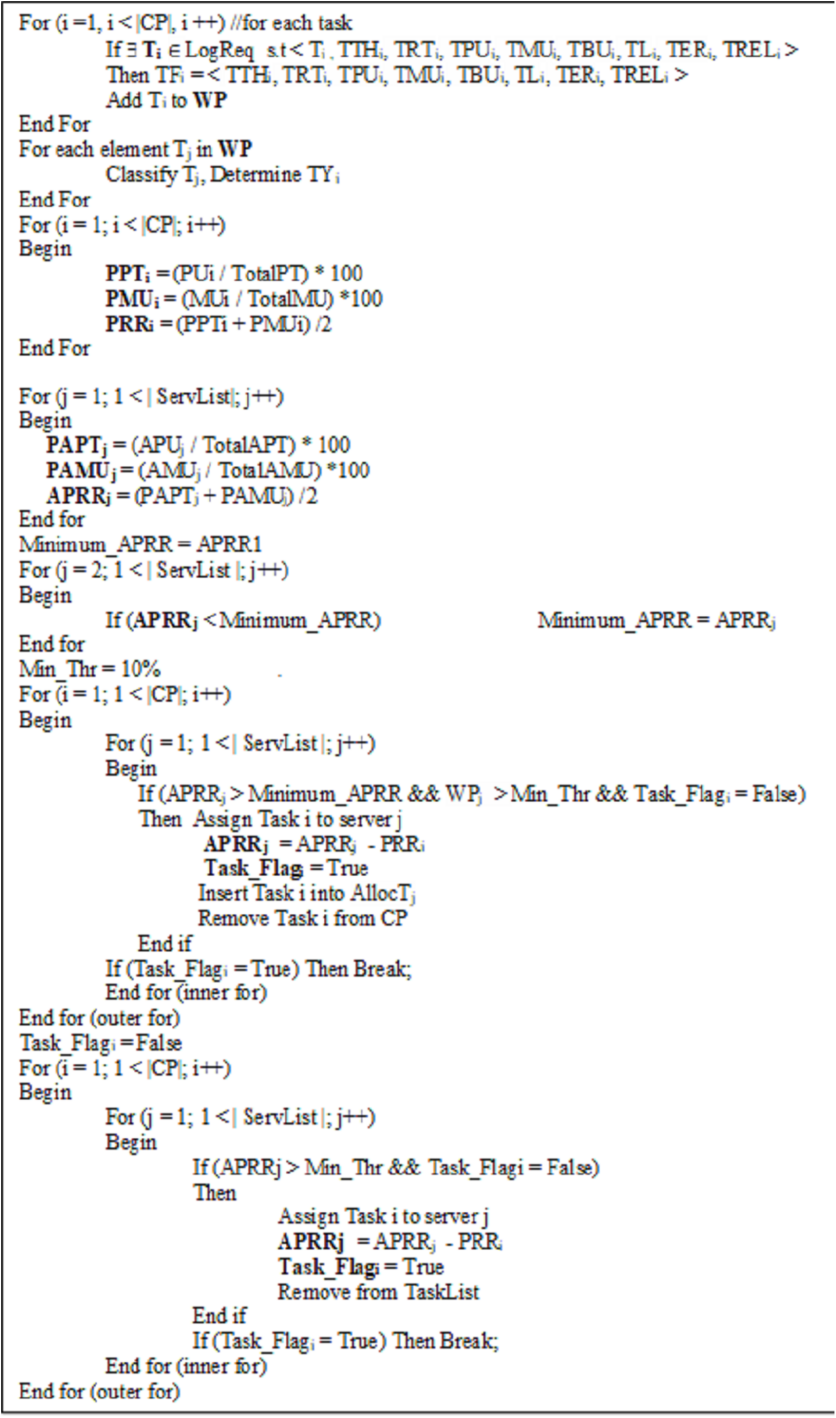
The pseudo-code for the proposed *Task Classification Load Balancing* algorithm.

The pseudo-code describing the *Task Classification Load Balancing* Algorithm is described in Fig. 2.

## Evaluation measures applied in the case studies

As discussed in “Background”, different parameters can be considered for evaluating the proposed algorithm. These parameters are presented in different research such as in Milani & Navimipour (2016) and Khedr, Idrees & Elseddawy (2016b) which include the following: “Algorithm nature, performance, flexibility, implementation, resource utilization, communication overhead, stability, adaptability, response time, reliability, complexity, and cost”. Ragsdale (2011) argued in his study that different attributes that describe the same entity may have a correlation relation between them. The attribute which has a strong relationship with other attributes is considered to be redundant. Therefore, although the previous set of parameters requires to be measured in order to ensure the applicability of the proposed algorithm as well as its advancement over other load balancing algorithms, however, the relations between these parameters can reduce the evaluation cost step as these relations ensure the effect of one parameter on another. In this section, the relations between the evaluation parameters are introduced, and then the set of parameters that will be included in the evaluation step are determined based on these relations.

***— Relation between response time, service time, transmission time and waiting time***

“*Response Time*” can be defined as the total required time for the server to perform the required service. It is the time interval between the service’s requests till the end of executing the service. Three-time intervals are involved, they are “*Service Time*”, “*Waiting Time*”, *and “Transmission Time”*. “*Service Time*” can also be defined as the required time interval to perform the required service. Moreover, “*Waiting Time*” is the time interval in which the service was waiting in the tasks’ pool without any assignment to any server. Finally, *“Transmission Time”* is the required time interval for the task to move from the tasks’ pool to the server. The response time is the total time produced by the summation of the three-time intervals, the request’s waiting time, service time, and transmission time. This reveals that if any of the three parameters increase, then the response time also increases. Therefore, the servers’ response time parameter can reflect the remaining required time parameters to perform the service. Response time is one of the parameters which will be used for the experiments’ evaluation in this study.

***— Relation between reliability, availability and downtime***

The system’s availability metric illustrates the total percentage in which the system performs its tasks relative to the total time. The total time describes the summation of the amount of downtime and the time in which the system is performing its tasks (Barker, Ramirez-Marquez & Rocco, 2013). Moreover, the system reliability metric illustrates the percentage of success for the system to accomplish the required tasks efficiently even in a limited factor (Pham, 2006). As discussed by Barker, Ramirez-Marquez & Rocco (2013), the availability of the system is directly affected by the system’s reliability. It is revealed by Barker, Ramirez-Marquez & Rocco (2013) that the reliability of the system increases the system’s availability which, consequently, reduces the system’s downtime. Based on this relation, this study measures the system reliability for the experiments’ evaluation in this study.

***— Relation between resource utilization, memory utilization, CPU utilization and network Bandwidth***

One of the performance measures is “resource utilization”. This parameter is divided into all the system’s resources such as CPU utilization, memory utilization, and the system’s bandwidth. The conducted experiments in this research provide a measurement of these resources.

***— Relation between performance, latency, bandwidth, throughput and response time***

According to (Alshaer, 2015), the performance of load balancing algorithms is measured through measuring different parameters including latency and throughput. The research in (Alshaer, 2015) presented different metrics for measuring the performance which included network bandwidth, throughput, latency, and fault tolerance with highlighting that the metrics that measure the performance can vary depending on the system's nature.

***— Relationship between fault tolerance, throughput, scalability and response time***

The research conducted in Sharma, Singh & Sharma (2008) discussed the relation between fault tolerance to be a direct negative relationship with the system’s performance. The fault tolerance metric presents the applicability of the system to adapt the system for continuing operating with the existence of failure in the network. Sharma, Singh & Sharma (2008) argued that fault tolerance has a direct effect on the system’s performance. If the system cannot recover from failure, then the system performance will directly be affected negatively. Moreover, as discussed in Sharma, Singh & Sharma (2008), performance is the main factor that can provide a clear view of the system’s scalability, the same research also provided that the main parameters for measuring the performance are the response time and throughput. In this research, the experiments’ evaluation included the average response time, minimum response time, and maximum response time as well as the number of hits per time unit.

## Simulation case study

Three experiments have been conducted to evaluate the applicability of the proposed algorithm. First, a simulation experiment has been conducted. A dataset was generated by (https://github.com/httperf/httperf) website. The dataset included 5,000 records; each record represented a task. The dataset is described by ten features. Four features are related to the task, they are type, size, processing time usage, and memory usage. The remaining six features describe the server, they are latency, response time, availability, throughput, success-ability, and reliability.

The authors used the StresStimulus; a load testing tool for web applications and cloud server performance. StresStimulus is a load-testing tool for websites as well as mobile and enterprise apps. It determines the web performance and scalability of the application under the rigors of heavy traffic load. Hundreds of thousands of physical users are realistically emulated through on-premises load generators or in a cloud-testing environment. At the same time, server monitoring information is collected in real-time to pinpoint application performance bottlenecks and isolate web speed issues. It’s an end-to-end test wizard that walks you through recording, configuring, and executing tests. The emulating workload included the following:

- Load Patterns: simulate production load, peak load, or run a stress test, set the number of VUs and select a steady or a step load pattern. If necessary, adjust the VU count after the test has started.
- The browser and the network mix: let the test client behave as hundreds of computers connected *via* different networks and using different browsers. Select browsers and network types from the list and add them to the mix. You can also add a custom network with a specific connection speed.

The evaluation of the “Task Classification” phase was performed using the five-fold validation approach. Determining the correctly classified tasks is applied by comparing between the task’s parameters and the cluster’s parameters in which the task is a member. The comparison was based on an acceptable range above and under the cluster’s parameters which guarantee a non-interference between clusters. This range is represented by the maximum and minimum values of the parameters which characterize the cluster’s members. For more clarification, all tasks have been run and their attributes have been measured. Then these tasks have been involved in the clustering phase, then the five-fold validation has been applied. 90% of the records that belong to each cluster have been considered as training data and 10% of each cluster have been considered as the testing data. The considered records have been altered for each fold so that it is included as training for all other folds. At the end of this process, each record should be involved in one of the iterations as a member of the testing dataset. Therefore, all members in the dataset are now having two labels, the original clustered label, and the predicted label after applying the enhanced ID3 algorithm. Then the evaluation process of the Enhanced ID3 algorithm (Khedr, Idrees & El Seddawy, 2016a) revealed the success of correct classification equal to 92.3% of the tasks with a total of 4,615 correctly classified tasks. The presented result reveals an error rate equal to 7.7% with a total of 385 incorrectly classified tasks.

After applying the proposed task classification load-balancing algorithm on the generated data, the performance of the system has been measured. The response time was equal to 2,588.5 ms, latency was equal to 39.2 ms, throughput was equal to 12.6 hits/s, and reliability was equal to 72.6. These results were further compared with five of the load balancing algorithms, they are round-robin, weighted round-robin, win-win, task scheduling, and bee colony algorithms. The comparison revealed that the proposed algorithm strongly competes with the other load-balancing algorithms due to its high performance which is illustrated in the evaluation measures. Table 1 presents the evaluation measures for the six algorithms.

**Table 1:** Evaluation measures results for the six load balancing algorithms.

Evaluation metric	Task classification	Round robin	Min-Min	Weighted round robin	Task scheduling	Bee colony
Response time (ms)	2,588.5	5,618.2	9,014.6	5,107.9	4,176.9	5,852.0
Latency (ms)	39.2	206.7	322.9	110.4	57.8	317.0
Throughput (Hits/second)	12.6	3.3	0.4	2.9	9.5	4.2
Reliability (%)	72.6	64.4	67.8	67.6	70.0	68.5

## Real case study applied in e-learning system and experimental results

The main aim of the applied experiment is to evaluate the users’ satisfaction represented in the students as well as the enhancement of the e-learning system targeting to support the educational field. The positive relation between the system performance and user satisfaction has been illustrated in different research such as in Lima et al. (2014).

In this study, two experiments have been applied in the e-learning system, which is the field on focus. The first experiment included 1,000 students while the second experiment included 5,000 students. The students belong to the faculty of commerce and business administration in Helwan University. The university is a governmental Egyptian university. The aim of applying two experiments with a different number of students is to confirm the applicability of the proposed algorithm during peak time. Both experiments have been evaluated by measuring the determined evaluation parameters which are discussed in “Evaluation Measures Applied in the Case Studies”. The learning system proposed five types of tasks representing the educational activities such as lectures’ videos, lectures’ notes, and performing exams. The students were eligible to perform five mandatory tasks and were requested to perform the assigned tasks in 60 min. No arrangement was required except for the online exam whose duration was 20 min as the last task. The authors used the Moodle system as an E-learning platform for the students, which is installed on Windows server 2008 R2 with the latest PHP version and SQL server 2008. All materials, quizzes, and assignments have been uploaded to the Moodle system.

The students are connected at different periods and under different tasks with different sizes. The authors used a rented cloud host for the experiment which had the following hardware and software configuration:

- Hardware configuration: 14 GB–24 GB RAM, 6 GB RAM Dynamic, 10 CPU Cores, 24 GHz Total CPU Power, 500 GB SSD Disk Space, Super Memory Cache.
- Network configuration: Unlimited Free SSL for Life (256 bits), VPN Support, Forex Optimized, Free CDN, CloudFlare Railgun, Unmetered Monthly Traffic, 1,000 Mbps Network Port, Uptime Guarantee, Unlimited Maximum Number of Web Sites.
- Software available: OS: Windows_2008_R2_Std, Crystal Reports Support, Webmail: Horde, Tomcat/Java Support, MS SQL Express, MS SQL, Oracle XE Support, MySQL Support, Microsoft Access, Automatic Backups (Snapshots), SolidCP Control Panel, Web Application Gallery, Classic ASP, ASP.NET 1.1, ASP.NET 2.0, PHP 4, PHP 5, Perl, CGI-BIN.

A comparison has been presented between the proposed algorithm and five of the well-known load-balancing algorithms. The comparison revealed to the higher impact of the proposed algorithm on the e-learning system performance. The idea of selecting algorithms of both static and dynamic categories is to reveal the advancement of the proposed algorithms on different mechanisms. The contributing algorithms in the comparison were round-robin, task scheduling, weight round-robin, min-min, and bee-colony.

Briefly, the task scheduling algorithm targets prioritizing tasks based on a defined criterion to reach higher resources’ utilization for the defined tasks in the appropriate time scheduling. The task scheduling algorithm allocates the tasks in a first-come-first-served strategy which has no criteria for assignment other than the available resources (Al-Maytami et al., 2021). On the other hand, the round-robin algorithm allocates tasks following the equal processor time distribution for the participating tasks using a time unit called “quantum”. The critical issue for round-robin is the good determination of the quantum duration time, which is the main pillar for its algorithm. Identifying incorrect large quantum time leads to raising the response time, while incorrect small quantum time leads to processor overhead. Consequently, identifying quantum duration may affect the task processing either to be completed or to be re-scheduled at the end of the queue to continue processing (Balharith & Alhaidari, 2019).

Moreover, the weighted round-robin algorithm provides weights for the services to identify the service’s requirements. Weighted round-robin divides the tasks into classes and then assigns fixed weight representing the quantum segments for each class. Although the algorithm is considered simple and low in computation, however, the fixed weight is not suitable for non-equal load tasks. This situation leads to lower throughput and poor performance in high traffic conditions (Saidu et al., 2014). Additionally, the min-min task schedules the required services in an ascending order based on their required execution time frame. That is the task with a required smaller time execution is served first. However, this strategy does not consider the machines’ load distribution and results in delaying the larger execution time tasks by preferring the tasks with lower time execution (Mathew, Sekaran & Jose, 2014). Finally, the bee-colony algorithm is used for scheduling tasks based on the foraging behavior of the honeybees which reflects the self-organization approach. It considers the previous nodes’ status while applying the load balance for the current nodes. As the bees announce the food representing the solution, therefore, the duration for the announcement raises the computation time.

### The experiments’ results (1,000 students, 5,000 students)

**Figure 3:**
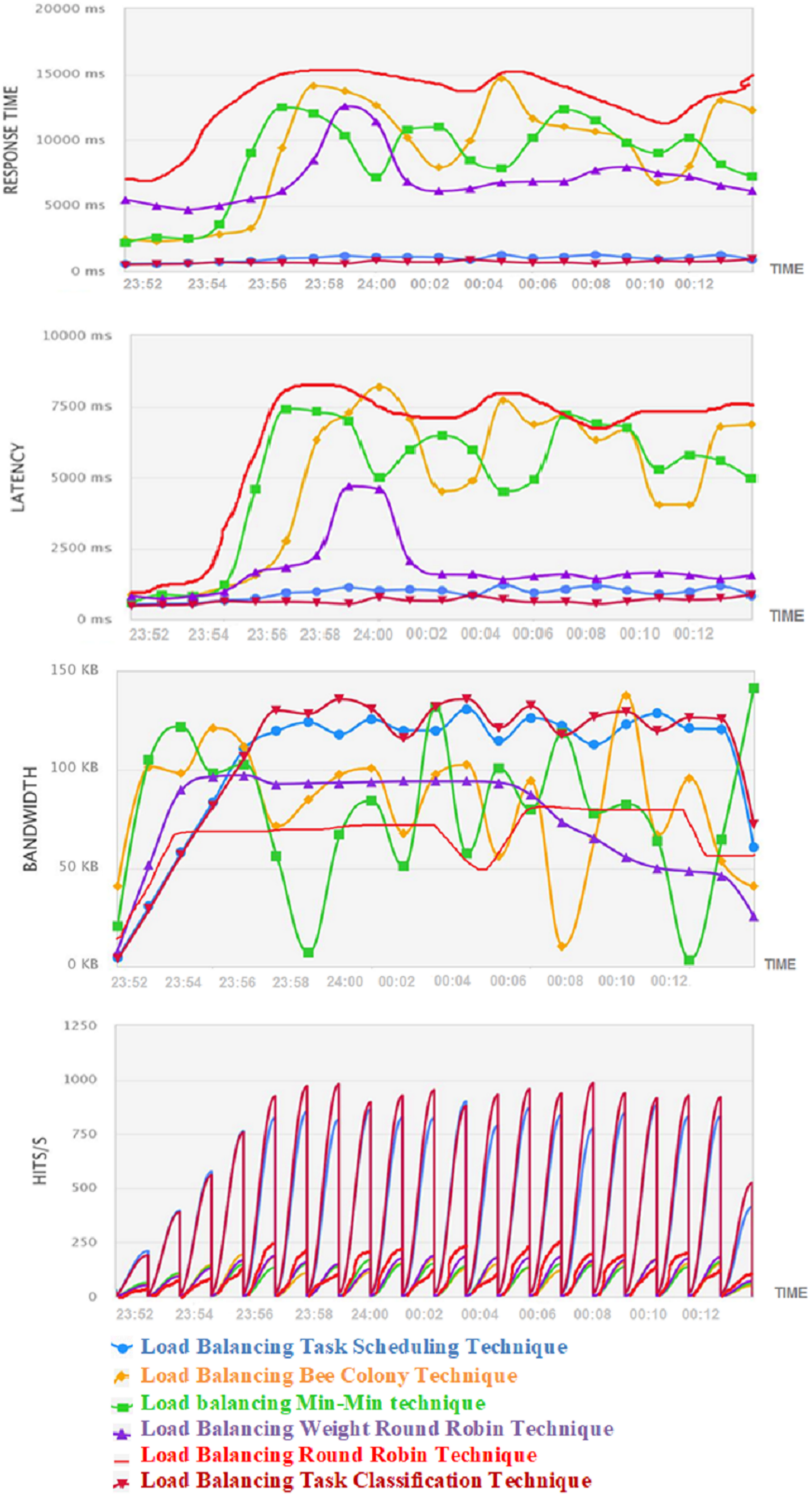
System monitoring comparison between five of the well-known algorithms and the proposed algorithm (experiment 5,000 students).

This section illustrates the experimental results of the proposed task classification load balancing algorithm for a system with a load of 1,000 active students performing different tasks in the first experiment and 5,000 students in the second experiment. The results also present the measures for the five mentioned load-balancing algorithms: round-robin, task scheduling, weight round-robin, min-min, and bee-colony. Table 2 presents the measures’ results for the algorithms under testing. Moreover, Fig. 3 demonstrates the monitoring of the system while using each of the six algorithms, the proposed algorithm (LBTC), bee colony (LBBC), min-min (LBMM), round robin (LBRR), weighted round-robin (LBWRR), and task scheduling (LBTS). The illustrated comparisons in both Table 2 and Fig. 3 confirm that the proposed algorithm provides the highest performance compared with other algorithms in the peak time. These results are further discussed in “Comments on the Experiments’ Results” and the behavior of the proposed algorithm is explained.

**Table 2:** Evaluation measures’ results for the algorithms (5,000 students).

Metric	Proposed task classification	Round robin	Task scheduling	Weight round robin	Min-Min	Bee colony
Avg. response time (ms)	4,870	10,570	9,740	9,610	16,960	11,010
Min. response time (ms)	1,229	1,242	225	1,909	6,764	1,217
Max. response time (ms)	70,111	60,061	8,033	19,911	31,005	138,342
Avg. latency (ms)	2,548.657	809.13	2,200.33	5,430.44	5,421.94	5,280.58
Avg. bandwidth (KB)	92.21	52.8	85.52	70.28	60.26	73.34
Avg. throughput (h/s)	9.88	2.58	7.45	2.27	0.3	3.29
Speed (ms)	6.77	5.25	5.9	4.45	3.48	5.87
Reliability	73.45	65.22	70.87	68.42	68.66	69.33

### Evaluation of the users’ satisfaction

The target of the proposed study is to enhance the e-learning system targeting to increase the users’ satisfaction level, which is considered one of the most important targets for using e-learning systems. A comparison has been applied between the e-learning system after applying the proposed algorithm and the e-learning system which has been developed in Khedr & Idrees (2017b). The reason for comparing these systems is that both of them have been applied in Helwan University, which provides a clear focus of the enhancement perspective.

Measuring users’ satisfaction has been on focus for a long time, therefore, scales have been proposed for this target. In Omar (1993), a user satisfaction scale has been developed which included 23 items that were following five factors. However, the only factor named “Information Quality and Accessibility” is considered in this study. Other scales have been proposed which focused on the same factors such as in Tojib & Sugianto (2007). The research considered the same factors for B2E products, the proposed scale was applied in Sugianto & Tojib (2007).

The main focus of this study is considering the factor of Information Accessibility. Therefore, this study has developed a review containing seven questions to measure the seven items representing this factor. The students provided two separate answers for each question, one for the previously applied e-learning system (Khedr & Idrees, 2017b), and the second answer for the proposed e-learning system. Therefore, two columns are provided for the questions, the first column before applying the proposed algorithm, and the second column after applying the proposed algorithm. A comparison has been performed for the students’ satisfaction level in both systems. The target of this comparison is to ensure the increase in the students’ satisfaction level when using the proposed system in the perspective of “Information Quality and Accessibility”. Seven measures have been included in the questionnaire, which are the system’s timeliness, system availability, the flexibility of data, user confidence in the system, system’s ease of access, system’s ease of use, and system’s reliability.

The provided review follows the five-point Likert scale that is introduced in Likert (1932). The five-point scale is presented for the reviews’ questions individually. The student determined his satisfaction level considering the question topic by selecting one value 1 which represents strongly disagree to five which represents strongly agree. Analyzing the results of this review is performed using a statistical method to present the student’s satisfaction level. The review has been distributed to the students and 3,670 out of 5,000 responses have been collected, the results of the review questions are demonstrated in Table 3. The results revealed an increase in the students’ satisfaction level considering the system’s performance.

**Table 3:** Results of evaluation measures for the algorithms.

Item	Proposed E-learning system		Previous E-learning system (Khedr & Idrees, 2017b)	
	Count	%	Count	%
Timeliness	3,464	95.37	3,272	89.16
Availability	3,500	96.37	3,119	84.99
Quick flexible access to data	3,475	94.69	3,096	84.36
Flexibility of data and reports	3,461	94.3	3,270	89.11
User confidence in system	3,509	95.6	3,370	91.85
Ease of access to system	3,570	97.72	3,091	84.23
Ease of use	3,430	93.46	3,304	90.05
**Average**	**3,450**	**95.36**	**3,217**	**87.66**

### Comments on the experiments’ results

It is revealed in the experiment’s results that the proposed task classification load balancing algorithm has the lowest response time. As previously discussed in “Evaluation Measures Applied in the Case Studies”, response time is related to the waiting time, transmission time, and execution time. Based on the proposed algorithm’s approach, the waiting time is minimized as the tasks are classified and then allocated in groups, not in sequence, based on the classification results which leads to a lower waiting time to the tasks in allocation as well as lower total transmission time. The execution time is also minimized as the successful allocation to the cloud nodes that are less utilized leads to a higher performance of the cloud nodes which supports faster execution of the required tasks. It is also revealed in the evaluation results that the task classification load balancing algorithm maintains the highest bandwidth of the system. Bandwidth is measured in bits/second, which represents the allowed amount of data that migrated from one point to the other through the network in the time unit. As the proposed algorithm has the lowest response time and, consequently, the lowest transmission time leads to more user requests being allowed in the system and migrate through the network for execution. The higher considered users’ requests in a time unit lead to the higher availability of the bandwidth.

Moreover, the throughput is measured by the number of hits per second-time unit. These hits represent the users’ requests. This means that the higher number of hits in a second reveals that the system can accept a larger number of requests. The system becomes able to accept the hits when it has available resources and available bandwidth. Therefore, based on the minimum response time of the proposed algorithm and the high bandwidth availability, this leads to the availability of the network to receive a higher number of hits. Additionally, the latency metric demonstrates the delay that the system may face during the operation. It is clear that lower latency is required to avoid bottlenecks in the network. The evaluation measures revealed that the proposed system provides the lowest latency among all the compared systems. This result is due to the low response time which grants the availability of the system to respond to more requests. This fast response normally leads to a lower latency of the system.

Finally, focusing on the algorithm complexity, the research in Ganeshan, Srinivasalou & Kousick (2013) reveals that the linear complexity of the round-robin and weighted round-robin algorithms to be O(1) which is the lowest complexity, however, it is discussed earlier in “Background” that static algorithms are less efficient, while Desai & Prajapati (2013) revealed the quadratic complexity of the min-min algorithm. Following the big O notation in calculating the algorithm complexity, it is deducted that the proposed algorithm is also following the linear complexity paradigm which contributes to the proposed algorithm due to its acceptable complexity and higher performance.

## Conclusion

This study proposed a novel load balancing algorithm that can be efficiently applied to the cloud environment. The target of applying the proposed algorithm is to enhance the e-learning system by ensuring the students’ highest satisfaction degree. This target is reached through optimizing the performance of the proposed algorithm in the e-learning process in general and in the peak time in specific.

The proposed algorithm was based on two main phases, the first phase targeted to classify the users’ requests based on the required resources and the predicted system’s performance. Then the second phase targeted the efficient requests’ allocation to the servers while maintaining the system’s performance. The allocation was based on selecting the lowest utilized server with respect to the highest utilized server as well as retaining the lowest utilization threshold. The allocation methodology aimed to avoid the servers’ over-loading and ensure a balanced load distribution.

The study proved the efficiency of the proposed algorithm through two main experiments. The first experiment was a simulation-based experiment that aimed to confirm the applicability of the classification phase and its impact on the whole process by evaluating the algorithm’s performance. The second experiment was a real-case experiment, the system was applied in Helwan University in Egypt which included two sub-experiments, 1,000 and 5,000 students. The results revealed the positive impact of the proposed algorithm on the e-learning system which is deployed on a cloud environment. The performance of the system was evaluated through the standard metrics which revealed satisfying results. The users’ satisfaction is also evaluated through distributing a questionnaire to the students. the questionnaire followed a standard user satisfaction scale to determine their satisfaction degree. The results revealed a satisfaction percentage equal to 93.9%. The proposed algorithm was compared with well-known algorithms to prove its advantages over these algorithms which followed different approaches, these algorithms are min-min, round-robin, weighted round-robin, task scheduling, and bee colony algorithms.

The future plan of this research is to apply the proposed algorithm in different domains, as well as examining the applicability to dynamically determine the suitable classification algorithm based on the field under examination. As different fields usually have different tasks to be applied, then determining the suitable classification algorithm will certainly raise the accuracy degree of the classification results. Moreover, the dependency between the tasks could be further considered for enhancing the proposed algorithm.

## Supplemental Information

10.7717/peerj-cs.669/supp-1 Supplemental Information 1 Dedicated Cloud Server Specification and Performance measurement method by the windows performance monitor.

10.7717/peerj-cs.669/supp-2 Supplemental Information 2 Questionnaire Items.

10.7717/peerj-cs.669/supp-3 Supplemental Information 3 Dataset.

10.7717/peerj-cs.669/supp-4 Supplemental Information 4 Students Questionnaire Response.

10.7717/peerj-cs.669/supp-5 Supplemental Information 5 requests code for measuring the system performance. the file includes the functions for measuring the system performance. it includes the modules for pre and post sending the requests' modules

10.7717/peerj-cs.669/supp-6 Supplemental Information 6 Create a connection to a given URL using GET method.

10.7717/peerj-cs.669/supp-7 Supplemental Information 7 Send the HTTP Get Requests to the blazemeter URL.

10.7717/peerj-cs.669/supp-8 Supplemental Information 8 Send the HTTP Post Requests to the blazemeter URL.

## Additional Information and Declarations

### Data Availability

The following information was supplied regarding data availability: Data and code are available in the Supplementary Files.
