# Supplementary material for: Enhancing the e-learning system based on a novel tasks’ classification load-balancing algorithm
Source: PeerJ Comput Sci. 2021 Sep 9;7:e669. doi: 10.7717/peerj-cs.669 (PMC8444070; doi:10.7717/peerj-cs.669)
Supplement: Supplemental Information 1 [file peerj-cs-07-669-s001.pdf]

Performance has been measured by the windows performance monitor

VPS Dedicated Cloud Server (Mocha Host)

- Dedicated Cloud
- **14 GB - 24 GB RAM** Guaranteed
- **6 GB RAM** Dynamic
- **10 CPU** Cores
- **24 GHz Total** CPU Power
- **500 GB** SSD Disk Space
- **Unlimited Free SSL for Life** (256 bit) δ
- VPN Support
- Forex Optimized
- Free CDN δ
- CloudFlare Railgun γ
- Unmetered Monthly Traffic
- **1,000 mbps** Network Port
- Uptime Guarantee
- LifeTime Discount®
- 180 Days Risk Free
- GDPR compliant
- SpamExperts - EGP47.00/mo
-
- Crystal Reports Support
- Webmail: Horde
- Tomcat/Java Support
- MS SQL Express
- MS SQL
- Oracle XE Support
- MySQL Support
- Automatic Backups (Snapshots)
- SolidCP Control Panel (Free)

- from EGP157.20/month *
- **Free Migration Assistance** α
- Let's Encrypt Support δ
- 1 LifeTime FREE Domain *
- **Super Memory Cache**
