# Supplementary material for: Enhancing the e-learning system based on a novel tasks’ classification load-balancing algorithm
Source: PeerJ Comput Sci. 2021 Sep 9;7:e669. doi: 10.7717/peerj-cs.669 (PMC8444070; doi:10.7717/peerj-cs.669)
Supplement: Supplemental Information 2 [file peerj-cs-07-669-s002.pdf]

System Timeliness
**State If the Following Actions Respond in an Acceptable Time**
Login to the system
Loading the new page
Transferring to the next page
Transferring to the previous page
Buttons’ response
Loading the summary page
**System’s Availability**
The server is available
The system is available in web browser
The system can be accessed using computer
The system can be accessed using mobile
The system can be accessed anytime during the day
The system can be accessed during peak durations (exam duration)
**Flexibility of Data**
Data could be viewed in different format
Data could be viewed in different views
Data could be viewed accumulative or individual
Slicing and dicing is available for headers
Using the browser facilities is available
**User Confidence in the System**
You trust that you can easily return to any page
You trust that the system will not fail
You trust that the system provides the required data
**System’s Ease of Access**
System registration is easy
System user data recoverability is easy
System can be accessed using different methods
**System’s Ease of Use**
Pages have a friendly interface
Pages have more than one method to access the required data
Pages’ contents can be easily understood
**System’s Reliability**
The system does not fail during peaks
The system performance is the same during peaks
The system pages are continuously available during peaks
The system can serve high number of users simultaneously
The system is secured against intruders
**Speed**
The web main page loads in an acceptable time
The migration from a part to another part is performed in an acceptable time
Loading data time is acceptable
Saving data time is acceptable
Generating reports time is acceptable
