# Supplementary material for: Enhancing the e-learning system based on a novel tasks’ classification load-balancing algorithm
Source: PeerJ Comput Sci. 2021 Sep 9;7:e669. doi: 10.7717/peerj-cs.669 (PMC8444070; doi:10.7717/peerj-cs.669)
Supplement: Supplemental Information 5 — the file includes the functions for measuring the system performance. it includes the modules for pre and post sending the requests' modules [file peerj-cs-07-669-s005.pdf]

Java HTTP Request using the built-in Java class *HttpUrlConnection*.

Http URL Connection class from **java.net** package is used to send Java HTTP Request programmatically by send **GET** and **POST** requests and then print the response.

The *HttpUrlConnection* class allows us to **perform basic HTTP** requests without the use of any additional libraries. All the classes that we need are part of the *java.net* package.

1- Creating a HTTP Request

We create an HttpUrlConnection instance using the openConnection () method of the URL class. This method only creates a connection object but doesn't establish the connection yet.

The *HttpUrlConnection* class is used for all types of requests by setting the *request Method* attribute to one of the values: GET

```
1- URL url = new URL ("http:// https://www.blazemeter.com/");
2- HttpURLConnection con = (HttpURLConnection) url.openConnection ();
3- con.setRequestMethod ("GET");
```

Create a connection to a given URL using GET method

2- Sending the HTTP Get Request

```
1- HttpURLConnectionProgram.java
2- package com.mkyong.http;
3- import javax.net.ssl.HttpsURLConnection;
4- import java.io.BufferedReader;
5- import java.io.DataOutputStream;
6- import java.io.InputStreamReader;
7- import java.net.HttpURLConnection;
8- import java.net.URL;

9- public class HttpURLConnectionExample {

10- public static void main(String[] args) throws Exception {

11- HttpURLConnectionExample obj = new HttpURLConnectionProgram();

12- System.out.println ("Testing 1 - Send Http GET request");
13- obj.sendGet ();

14- System.out.println ("Testing 2 - Send Http POST request");
15- obj.sendPost ();

16-   }

17- private void sendGet () throws Exception {

18- String url = "https://www.blazemeter.com/";

19- HttpURLConnection httpClient =
20- (HttpURLConnection) new URL(url).openConnection();
   }
[illegible]
```

Send the HTTP Get Requests to the blazemeter URL

3- Sending the HTTP Post Request

```
httpClient.setDoOutput (true);
try (DataOutputStream wr = new
DataOutputStream (httpClient.getOutputStream())) {
 wr.writeBytes (urlParameters);
 wr.flush ();
  }
int resCode = httpClient.getResponseCode ();
System.out.println("\n URL Request " + url);
System.out.println("Post " + urlParameters);
System.out.println("Response " + resCode);
try (BufferedReader in = new BufferedReader(
new InputStreamReader (httpClient.getInputStream()))) {
String rline;
StringBuilder response = new StringBuilder();
while ((rline = in.readLine()) != null) {
response.append (rline);
}
//print result
System.out.println (response.toString ());

}

}
```

Send the HTTP Post Requests to the blazemeter URL

HTTP GET

The method “HTTP GET” requires the determined resource’ description. The result of this method is retrieved data

HTTP POST

The HTTP POST method sends data to the server. It is often used when uploading a file or when submitting a completed web form.
